# Improving curriculum delivery: Using a results informed quality improvement model for teen behavioral health education

**DOI:** 10.3389/fpubh.2022.965534

**Published:** 2022-11-16

**Authors:** Gregory Gross, Rui Ling, Brad Richardson

**Affiliations:** ^1^National Resource Center for Family Centered Practice, University of Iowa School of Social Work, Des Moines, IA, United States; ^2^Brown School, Washington University, St. Louis, MO, United States

**Keywords:** continuous quality improvement, behavioral health education, three-stage (3S) quality improvement process model, participatory approach to improving instruction, curriculum delivery, improving outcomes of instruction

## Abstract

Adolescence is a critical developmental stage to establish healthy decision-making processes and behavior patterns. Many interventions such as evidence-based curricula have been implemented to guide adolescents to avoid risk-taking behaviors and improve health and medical knowledge and outcomes. This study presents a participatory approach informed by the three-stage (3S) quality improvement process model to improve the quality of curriculum delivery, based on the results indicating outcomes achieved, needs for improvement, and quality assurance for maintaining the expected outcomes of an evidence-based curricula. Tests were conducted before and after the intervention. Using threshold levels and measures of change in the tests, instructors participated in guided discussion and analysis of the data to identify where and how instructional improvements should be made and where outcomes were being achieved as expected. This method was used to diagnose variation in the results and delivery and identify root causes informing actions to improve curriculum delivery and outcomes. After the facilitated discussions, pre- and post-tests from subsequent classes were analyzed. The results showed improved test item scores ranging from 2 to 69.5% and seven of 18 items obtained statistical significance following the implementation of the model described. Overall, an increase in the mean percent correct of 17.1% was found.

## Introduction

Adolescence is an important developmental stage to establish healthy decision-making processes and healthy behavior patterns (1). However, according to the 2017 Youth Risk Behavior Surveillance (YRBS) Report (2), many high school students are engaged in risk-taking behaviors, such as unsafe driving, substance use, unprotected sex, and unhealthy diet, which are associated with premature mortality, morbidity, and social problems among persons aged 10–24 years in the United States (2). Adolescent use of tobacco in the United States, including nicotine-containing electronic vapor products, continues to increase in 2019 (3, 4). Sexual risk-taking behavior like unprotected sex or multiple partners relates to unexpected pregnancy, sexually transmitted infections (STIs), mental health, academic attainments (5, 6). From the YRBS report, only 53.8% of the respondents who are sexually active reported using a condom during their last sexual intercourse (2). Youth account for about 50% of the STIs cases in the United States (7, 8).

To help adolescents be aware and avoid risk-taking behaviors, numerous methods have been applied or discussed (6, 9). Some focused on school-based or group-based activities to promote risk avoidance (9–13), while others focused on relationships with parents or other trusted adults to affect decisions about risk-taking behavior (14–16). We also found digital intervention that aimed to target individual decision-making skills to promote healthy behaviors (17–19). Moreover, a variety of curriculum evaluation models were developed and used in the past few decades to look into the outcomes of the school-based education. A mini-systematic review in 2020 presents seven different models and frameworks for curriculum evaluation, including the CIPP Model, the Four-Level Model of Learning Evaluation, and Philips' Model of Learning Evaluation (20–23). The CIPP Model first developed by Stufflebeam has been used by researchers in a wide range of contexts worldwide, looking at the overall education process and outcomes (21, 24–26).

While extensive effort is involved with developing interventions and evaluation of the outcomes of curricula from a macro point of view, relatively little attention is given to the means of improving delivery process quality based on outcomes and assessment data (27). Quality improvement of intervention could be another trajectory to achieve the goal of improving adolescent health.

Quality improvement has been utilized in other fields for a long time, and the use of it in health instruction can be traced to at least the 1990s (28, 29). In healthcare, quality improvement was defined as a continuous process to improve the efficiency, effectiveness, outcomes, or other indicators of quality in a program, leading to achieving the aims of health equity and community health improvement (29, 30). Three essential features of continuous quality improvement (CQI) were identified in a systematic review, including “systematic data-guided activities,” “designing with local conditions in mind,” and “iterative development and testing process” (31). The benefits of CQI on improving the health outcomes remain unclear (28), but we do see positive outcomes from some of the CQI studies. Doherty et al. studied a participatory quality improvement intervention to improve the coverage of a mother-to-child transmission prevention program in South Africa which resulted in great improvements in the program indicators (32). Iyengar et al. reported substantial improvement in adherence to childbirth practices after implementing a quality improvement intervention in India (33).

This present study is a natural experiment with an iterative participatory quality improvement model designed to aid delivery of an adolescent behavioral health curriculum using pre- and post-tests. The purpose of the study is to demonstrate a CQI model using the results to inform curriculum delivery based on a multi-site implementation of a teen behavioral health education curriculum. The study was also designed to meet one of the goals for the Office of Population Affairs which was to increase the quality of program delivery intended to improve gains in student knowledge.

Powerful Choices (34) is a curriculum designed for school-based delivery to promote decision-making for healthy choices, avoid risky behaviors, and promote positive attitudes, protective factors, and behavioral intentions. The curriculum includes 10 sessions: wisdom, awareness, friendship, control, courage, knowledge, boundaries, excellence, ambition, and success. All curriculum instructors received training from the curriculum developer. The primary focus of the curriculum is to teach good decision-making and avoid risk behavior that could lead to negative consequences, including unintended pregnancy. For evaluation purposes, a survey was administered to students who participated in the classes before and after the curriculum. Knowledge of key content was gathered using an 18-item instrument, authored with the developer, piloted, revised, and tested for validity prior to implementation.

## Models and methods

### Three-stage quality improvement process model: Results, diagnosis, and focus for improving the results

**Figure 1:**
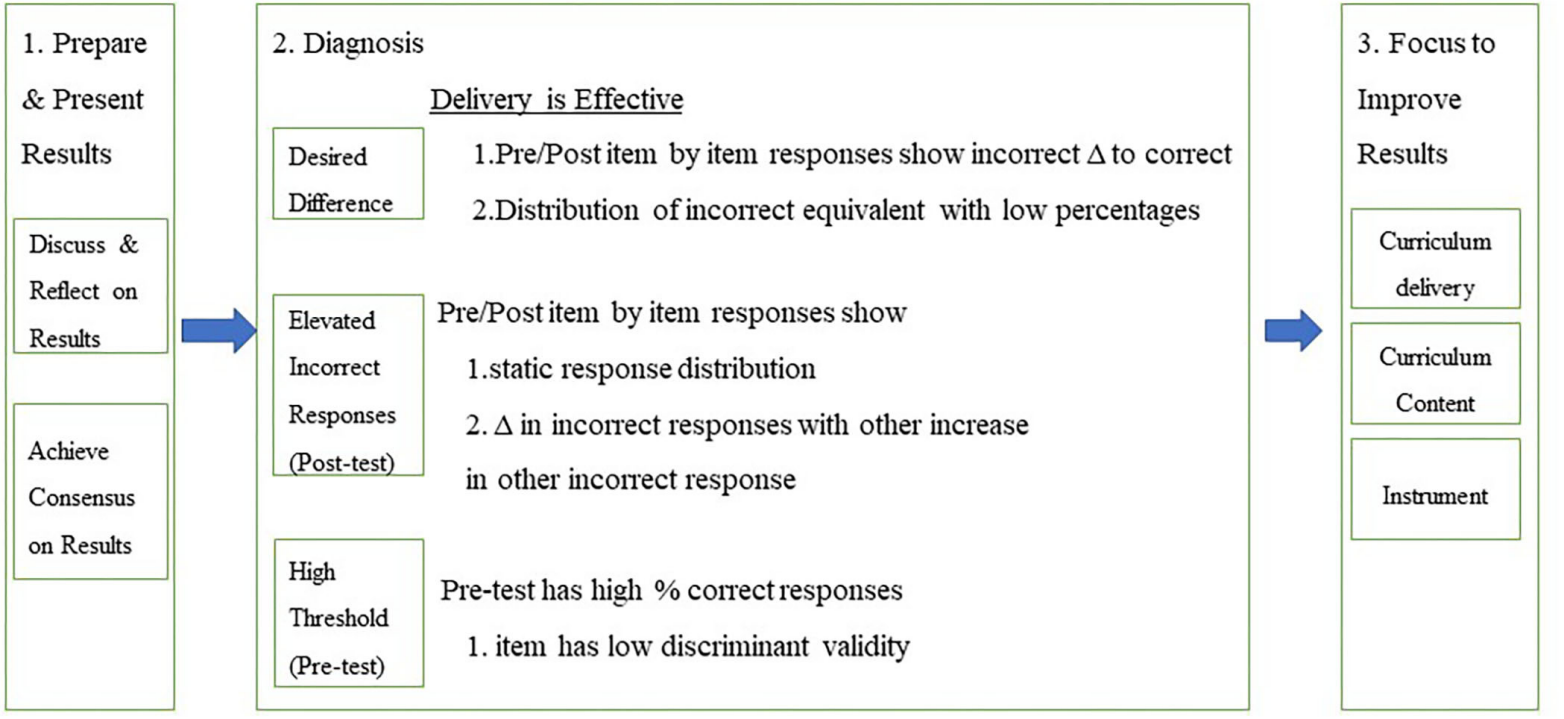
Three-stage quality improvement process.

The 3S quality improvement process model (Figure 1) was designed and used to improve the delivery quality of the Powerful Choices curriculum. It is a participatory CQI model that values the dissemination of the results to inform delivery improvement. The model includes three stages: preparing and presenting the results, diagnosis, and improving the results. The core element of the model is the qualitative facilitated discussion of the results and their interpretation held in the second stage, which provides the opportunity for skilled evaluators to facilitate reflective discussions of the results with instructors focusing on how the results “fit” with the classroom experience. The first stage starts before the discussion when the evaluation team prepares the results in a format that is understandable and easy to follow and performs preliminary analysis summarized in a brief written statement to guide the discussion (see, e.g., Tables 1, 2). This discussion should happen soon after the team has evaluation results available (e.g., in the week following the completion of instruction). During the discussion, the facilitator first describes the results and the summary of each item to make sure the instructors have a good understanding of what the data show. In the second stage, evaluators facilitate discussion to help diagnosis what might account for scores that are lower than desired guided by the three basic results scenarios which guide the identification of planned action. The three scenarios are described below:

- Scenario one: In a situation in which there is a high percentage correct at pre-test. Although the purpose of validating the instrument through the piloting process should lead to relatively low correct percentages at baseline (pre-test), a high percentage of correct pre-test answers may indicate that the content is generally known and a detectable difference at post-test would be difficult to obtain. It could also indicate that choices in the response set require revision because the correct answer is easily identified prior to participation in the delivery of the curriculum.
- Scenario two: The second commonly encountered result is higher than the desired number of incorrect responses due to static, or no change in responses (e.g., students “stick with” their original answer). This can be seen when the percentages at pre-test and post-test for each response are nearly the same. The discussion focuses on what may be happening in the delivery that is not clearly providing information consistent with the correct answer or maybe meeting resistance.
- Scenario three: The third scenario is where an incorrect response is chosen on the pre-test, and on post-test a different but incorrect choice is selected. This can be seen in a table where, for example, if 20% choose one incorrect response on the pre-test and on the post-test, a substantial number from the 20% consistently chose another incorrect response.

**Table 1:** Initial (pre-test) by follow-up (post-test) knowledge test item (Group 1).

	**To have the most successful life a person should?**					**Initial Totals**
**Initial**	**Follow-up**					
		**A**.	**B**.	**C**.	**D**.	
A. Finish high school, get a good job, not have children before marriage.	Count	67	5	7	4	83
	% of Total	38.7%	2.9%	4.1%	2.3%	48.0%
B. Remain single, go to college, get a good job.	Count	7	9	1	1	18
	% of Total	4.1%	5.2%	0.6%	0.6%	10.4%
C. Get a good job, pay all bills on time.	Count	21	2	4	6	33
	% of Total	12.1%	1.2%	2.3%	3.5%	19.1%
D. Get a job that I like and that pays well.	Count	23	3	7	6	39
	% of Total	13.3%	1.7%	4.1%	3.5%	22.5%
Follow-up totals	Count	118	19	19	17	173
	% of Total	68.2%	11.0%	11.0%	9.8%	100.0%

The follow-up assessment obtained 68.2% correct compared to the initial 48% correct for choice “A,” the correct response. The greatest improvement from initial assessment to follow-up was the choice “D” with less than half of the students who had selected this response initially choosing the incorrect response at follow-up. Choice “B” is the area of opportunity as 18 students chose the response incorrectly initially and 19 students (11.0%) chose the response at follow-up. In addition, while there was a decrease in the number of students selecting “C,” 19 students still chose the response incorrectly at follow-up.

**Table 2:** Initial (pre-test) by follow-up (post-test) knowledge test item (Group 2).

	**To have the most successful life a person should?**					**Initial totals**
**Initial**	**Follow-up**					
		**A**.	**B**.	**C**.	**D**.	
A. Finish high school, get a good job, not have children before marriage.	Count	80	4	1	0	85
	% of Total	44.9%	2.2%	0.6%	0.0%	47.8%
B. Remain single, go to college, get a good job.	Count	11	6	2	1	20
	% of Total	6.2%	3.4%	1.1%	0.6%	11.2%
C. Get a good job, pay all bills on time.	Count	21	0	3	3	27
	% of Total	11.8%	0.0%	1.7%	1.7%	15.2%
D. Get a job that I like and that pays well.	Count	34	1	0	11	46
	% of Total	19.1%	0.6%	0.0%	6.2%	25.8%
Follow-up totals	Count	146	11	6	15	178
	% of Total	82.0%	6.2%	3.4%	8.4%	100.0%

The follow-up assessment obtained 82% correct compared to the initial 47.8% correct for choice “A,” the correct response. The dramatic improvement in the correct responses at follow-up accompanies consistently low frequencies of incorrect responses across all incorrect response options at follow-up demonstrating an example of the desired improvement and suggests effective curriculum delivery.

After diagnosis, a more in-depth discussion is facilitated to discover the root cause based on the information at hand including the guidance offered by the scenarios from the diagnosis, identifying the source of the problems (35, 36). It starts from the problems diagnosed at the last step and continues with asking why it happened until the group reaches agreement about the root cause. Lastly, the discussion focuses on action planning^1^ to improve the results based on the information already discussed crucial for identifying underlying reasons including curriculum content, delivery process, and the instrument used for assessment.

### Procedures

During the 2018–2019 and 2019–2020 school years, the Powerful Choices curriculum was delivered in eight school districts. Students participating in the Spring lessons completed a knowledge pre-test in the beginning session of the Powerful Choices lessons and completed a knowledge post-test at the end of the last session (Group 1). Data were analyzed by the evaluation team, and a summary of the results was written for each set of pre- and post-test comparisons for the discussion with instructors and the curriculum developer. Evaluators guided the discussion using the three-stage (3S) quality improvement model (Appendix 1). A table for each of the 18 questions on the pre-test and post-test was produced to show the number and percentage for each response category on each test item in a pre-test by post-test table. Highlighted rows (pre-test) and columns (post-test) provide data visualization of correct answers for easier interpretation. Each table included a brief narrative describing the distribution of responses including meaningful changes observed among response items for each question.

Following the completed delivery of instruction in Spring 2019, the facilitated discussion was held with five instructors and the curriculum developer to review the results and identify strengths, and areas for improvement based on the data. The discussions were facilitated by the evaluation team guided by the 3S quality improvement model:

- Results: review tables and narratives, facilitated discussion, and consensus on the results.
- Diagnosis: using three results scenarios to guide decision-making about the results.
- Focus to improve results: root cause analysis and consensus (curriculum content, curriculum delivery, and instrument) determining actions for improvement (see footnote 1).

The facilitated discussion takes about 1 h. During the discussion, the facilitator started with a brief introduction about the purpose and the agenda of the meeting, followed by the discussion of each of the 18 sets of questions. For each question, the facilitator first reviewed with instructors the tables and brief narratives describing the comparative results in the table showing the pre- and post-test results by response selected and percent correct at pre- and post-test. Next, the facilitator allowed for some reflection on the results and used the three scenarios in the diagnosis portion of the model to guide the discussion of reasons for changes on each item. For those items with the post-test results of <70% correct, the discussion focused on the root cause and corrective action that could improve the delivery process so that students could better understand the curriculum content. The instructors started classes in the 2019–2020 school year with the planned actions. Another group of students participated in the Powerful Choices Fall classes during the 2020–2021 school year as referred as Group 2. The same pre- and post-tests were administrated before and after the classes. The comparison analysis of the two groups was done after the classes.

### Data

Data were collected using student input into an electronic survey application at the conclusion classroom delivery of Powerful Choices in eight midwestern school districts. The eight school districts were similar in size, location in the state, and general characteristics of students attending. Students were asked to take the 18-item knowledge test prior to the beginning of the first instructional session (pre-test) and at the conclusion of the final instructional session (post-test). Parental permission was obtained by the program for student participation in the program and study. Tests were de-identified by the use of a respondent code known only to the student and school district teacher (not the curriculum instructor). The current analysis used a de-identified dataset and given the nature of identity protection and data security, The University of Iowa Institutional Review Board determined that the project did not meet the federal definition of human subjects' research and issued a Human Subjects Research Determination letter to that effect.

### Measures

#### Demographics

Demographic information included sex, grade level, school, race, and ethnicity.

#### Curriculum knowledge

An 18-item (Appendix 2) knowledge test was designed with the curriculum developer based on key curriculum content. Students could answer the 18 questions by selecting one of four response options for each question. The validity and reliability of the instrument were tested with the developer as the trainer. Testing was conducted based on a pilot set of four classes. The instrument was revised with the curriculum developer and tested a second time with a different set of classes. Refinement of the instrument was based on internal consistency reliability and percent correct for each knowledge content item. The post-test instrument included satisfaction items and was otherwise identical to the pre-test.

### Analysis

Data were analyzed using SPSS 27. Crosstabulation tables were created to compare pre-test to post-test responses for each item for each of the two groups. Tables were post-processed to highlight the row (pre-test) and column (post-test) with the correct answer. Missing data were excluded by test item (“pairwise deletion”). To determine whether significant differences were obtained, correct responses were coded 1 and incorrect responses were coded 0 resulting in the mean score and the percent correct being virtually the same number. *T*-tests were calculated to determine the statistical significance of differences. Of interest to the program was the achievement of 70–80% correct at post-test; therefore, we also examine the percentage of correct responses on the post-test.

## Results

### Three-stage quality improvement process

To prepare for the facilitated discussion, pre-test by post-test tables of responses for each of the 18 items of the instrument were created for review. A typical example of information provided to instructional staff for the facilitated discussion is presented in Table 1 below. The column labeled Initial Totals (far right column) shows the number and percentage of participants choosing each answer at pre-test. The row labeled Follow-up Totals (bottom row) shows the number and percent choosing each answer at post-test. The correct response is highlighted (e.g., Answer A in Table 1) for each test question. Overall, 118 (68.2%) chose A on the post-test compared to 83 (48.0%) who chose A on the pre-test; this is an increase of 20.2%.

With the presentation of the results to the instructors, the evaluation team facilitated discussion of each question comparing pre-test and post-test responses. Following the discussion of the results and achieving a consensus or common understanding of those results, instructors were asked what they thought could account for the change in responses, and what could be improved to achieve a higher percentage of correct responses (i.e., change of delivery, curriculum content, or test question and response items). The discussion of each test question and responses followed this general procedure. The discussion of all 18 items took about 1 h.

To provide an example of the discussions that take place, we provide a typical discussion that took place leading to the identification and adoption of strategies for improvement.

*Example from one facilitated discussion:*

*Evaluator: For this question there was a 20 percent increase in correct responses at post-test. However, 32% still chose one of the incorrect answers. While the results show improvement in number and percent of correct responses between pretest and posttest, the overall percent correct at post-test is still lower than the conventional target of 70–80% target correct. What might account for difference and how could it be further improved?*

*Instructor 1: Well, that's one of the lessons that I struggle with, students don't seem to grasp that part as well as other lessons*.

*Instructor 2: I agree. They seem to come in with a lot of preconceived ideas that are hard to change with our instruction*.

*Instructor 3: Exactly! The incorrect answers they chose are not necessarily wrong, it's just that they are not all are relying on what was taught but their attitudes about things that they held before attending the class. That isn't the best way to build a strong foundation for your life*.

*Evaluator: That's a good point. Looking at the results, there are about 11% who stayed with the same answer even though it was not the correct answer: 9 (5.2%) stayed with B, 4 (2.3%) stayed with C, and 6 (3.5%) stayed with D. Thinking about the possible reasons, sources or causes of these results, would you say it is more due to the curriculum, or more due to how it is being delivered?*

*Instructor 1: Oh, this one definitely fits in curriculum delivery. I know there's got to be a better way to present this material, so the students understand that not having children before marriage is a key to finishing school and getting a good job*.

*Evaluator: Is there a similar challenge where you have discovered students not completely getting what you're telling them, and it shows up later? What have you done and how might that apply here?*

*Instructor 2: Similar to one of the one we talked about earlier questions we talked about being sure to say it a second time and ask a question so that students say the words together so that is “sticks.”*

As shown from the example conversation above, the evaluator facilitated discussion helping guide the instructors in exploring the reasons why any specific item may have been challenging for students based on the test results. The table of the results made it easy for instructors to see the responses and offer their perspectives from their experience teaching the students. In the example, two specific reasons were identified: (1) students come into the class with preconceived ideas that are hard to change; and (2) difficulty choosing the one correct answer according to the curriculum when the other options are not necessarily wrong, but less relevant. To address the problem, instructors proposed to enhance students' understanding and memories related to the questions by repeating the curriculum content. Also, having more interaction with students during the lectures to help students grasp the main idea the question was capturing that “not having children before marriage is a key to finishing school and getting a good job.”

### Comparison of two groups

A total of 351 7th- and 8th-grade students participated in Powerful Choices and completed the instruments. Of the 173 students in Group 1, 55.5% were boys, 52% were in 7th grade, and 91% were white/Caucasian. Of the 178 students in Group 2 who participated in Powerful Choices classes in Fall, 2019 (after the facilitated discussion and improvement strategies were implemented), 53.9% were boys, 47.8% were in 7th grade, and 94%, were white/Caucasian.

Table 2 presents the results from the same question presented in Table 1 (To have the most successful life a person should?), but the results in Table 2 reflect the adjustments to instruction made in delivering the curriculum to Group 2 based on the facilitated discussion from Group 1. The percent correct at pre-test for Group 2 in Table 2 (47.8%) is nearly identical to the percent correct for Group 1 in Table 1 (47.9%) indicating similar knowledge levels at pre-test; however, the percent correct at post-test increased to 82% for Group 2 compared to 68.2% in Group 1.

**Figure 2:**
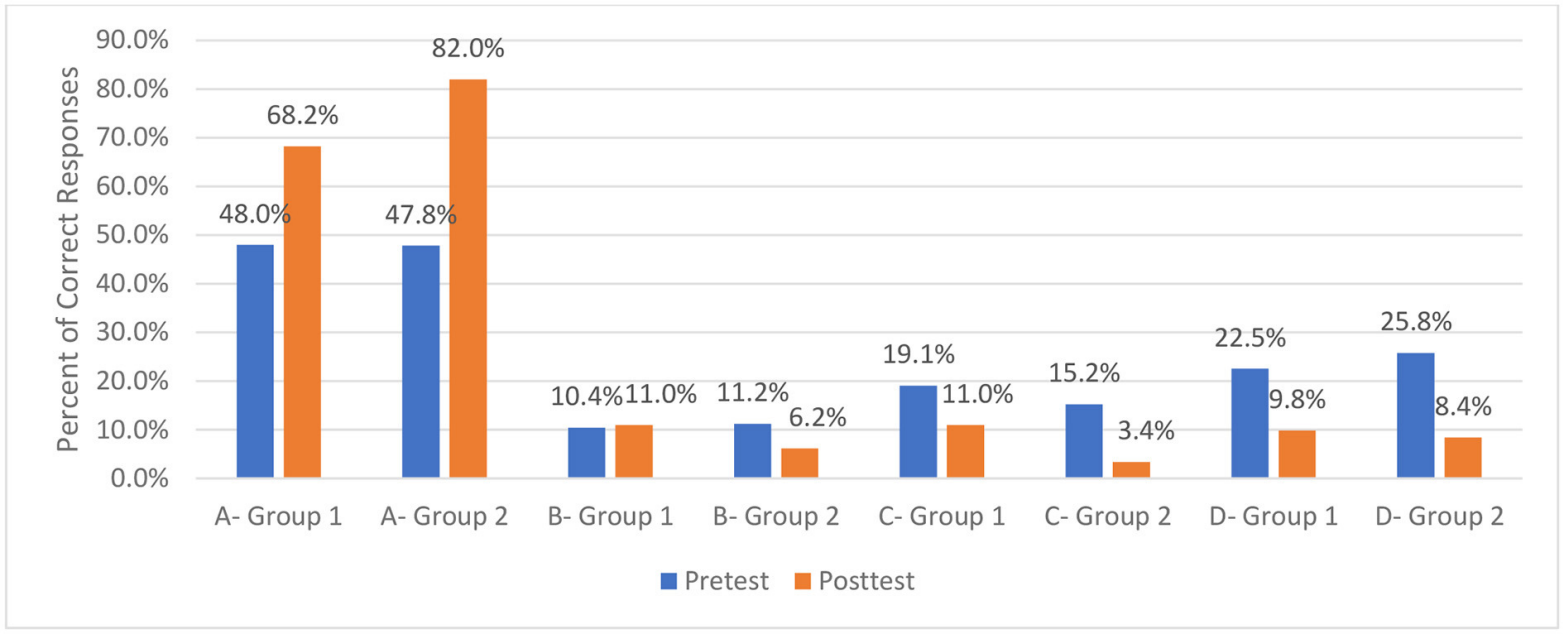
Percent corrected by response category (Group 1 compared to Group 2). Response categories for two groups (Group 1 Response A, Group 2 Response A, Group 1 Response B, Group 2 Response B). Response A is the correct answer.

Figure 2 illustrates the distribution differences for each of the response categories (Table 1 compared to Table 2; Group 1 is labeled Cohort 1, and Group 2 is labeled Cohort 2). Comparison of percentages for Response A, the correct answer, is typical of what many programs use as the only measure of assessing effectiveness (percent correct); however, such comparisons do not provide the level of detail needed to assess specific response by response “movement” from pre-test answer to post-test answer that the tables we use provide.

The analysis of the results shows changes for each response (pre-test and post-test) on the instrument for those participating in classes before adjustments were made based on the facilitated discussion, and after adjustments were made before delivery of the curriculum to Group 2. The results of the improvement across all 18 items of the instrument are presented in Table 3. Table 3 presents the percent correct at post-test by question for the two groups. Overall, increases in the percent of correct responses at post-test were found on 16 of the 18 items. Statistically significant improvement in the percent correct was found for seven items (*p*. ≤ 0.05). Perhaps more importantly, at post-test for Group 2, 10 items reached a 70% correct threshold, and seven items reached an 80% correct threshold.

**Table 3:** Percent correct at post-test by group and *t*-test of significance between groups.

	**Percent correct, Group 1**	**Percent correct, Group 2**
**Instrument questions**	**(*N* = 173)**	**(*N* = 178)**
K1. When I need to make an important decision I would?	61.9	72.7
K2. Gaining positive relationships is experienced best by?	13.1	82.6^*^
K3. The best way to develop a close friendship is?	68.0	70.2
K4. How do I avoid behavior that could have a negative consequence for me?	74.9	80.9
K5. What makes people successful in life?	73.0	86.5
K6. How can you avoid bad habits?	53.4	94.9^*^
K7. What would you do if a person from your class spread a rumor about your close friend on social media?	28.7	64.6^*^
K8. Wisdom is best gained by which of the following?	59.1	91.6^*^
K9. According to Powerful Choices lessons, when is the best time for a person to become sexually active?	30.7	46.3
K10. Accepting the challenge of thinking before acting helps a person to?	35.3	52.0^*^
K11. How can you build a strong foundation for your life?	63.0	67.6
K12. The use of drugs and alcohol have the strongest effect on?	57.7	63.1
K13. What does a person need to do to make good decisions for the future?	56.8	65.7
K14. Making a good decision is a result of?	74.3	77.3
K15. Taking Powerful Choices lessons results in?	13.4	80.9^*^
K16. To have the most successful life a person should?	68.2	82.0^*^
K17. How do you show your friend that you care when they make mistakes?	69.1	48.3
K18. How can you be a positive role model?	60.6	42.1

* p. < 0.05.

## Discussion

In this study, we introduce a three-stage process using tables of responses to questions before (pre-test) and after (post-test) participating in a course of instruction using a curriculum titled Powerful Choices. The results demonstrated improvement on the post-test results among participants following the facilitated discussion of the results with instructors. Taken together, the results from this study demonstrate an effective approach for improving curriculum delivery and using the results to engage instructors in examining how they may contribute to achieving improved effectiveness for learning content by students in their classes. The analysis of root cause discussions leads to one of three categories for revision: curriculum delivery, curriculum content, or the instrument measuring knowledge (attribution to the instrument tends to fade as an explanation after the instrument is piloted and used in practice a time or two). The model was designed to be an iterative process because of the reality of drift in curriculum delivery fidelity and effectiveness. The model is a reset strategy that is engaging and improves fidelity and effectiveness. Using the three-stage quality improvement process, program evaluators and instructional staff working together are well-positioned to track the results and use the model to facilitate the discussion for ongoing assessment of effectiveness and improvement in curriculum delivery. Programs early in their development will identify more revisions. As programs develop and mature, the curriculum and testing become more “standardized” as part of the organizational culture. Delivery may stand out as the primary mechanism for improvement as programs mature; however, the discussion of causes and strategies for improvement remains key for highly effective instruction.

The results suggest that the use of full information from responses and using pre-tests and post-tests, not post-tests alone, is important for identifying factors to consider in the discussions of root causes and is an effective approach to improve curriculum delivery. Deming emphasized that it was important to study, reflect on the data, and from that take actions to improve the program (27). The process is one of a continuous feedback spiral toward continuous improvement. The process described here is participatory involving instructional staff. It is also highly efficient in terms of time and engages all involved in data-guided discussion to focus instructors on their accounts of what may underlie the results and how instruction could be adjusted to improve the results. In fidelity monitoring, observations are typically only conducted on a subset of lessons, and the focus is on the process, not the results. The model described in the present study helps instructors see across all of their lessons, helps illuminate blind spots that may exist, and engages and assists instructors in identifying what modifications could best improve curriculum delivery.

### Limitations

The positive results support the benefit of using the three-stage quality improvement model. The results were derived from a natural experiment without the benefit of a comparison or control group which is necessary for more robust findings. Without the benefit of a control group, the effect of other factors that could account for improvements in knowledge scores is not known. Also, given the nature of the natural experiment, notes rather than verbatim documentation of the discussions providing qualitative data were identified as a limitation. An example of the discussion was presented to demonstrate an example of the way in which the discussion was guided. Further investigation using a more rigorous study design and documentation, and preferably a randomly controlled trial to replicate the process, is needed to further support the model by comparing the results under the two conditions.

## Conclusion

The results of the present study suggest the three-stage quality improvement process model used to improve the quality of the Powerful Choices curriculum is feasible and effective. It is a practical, data-driven approach that enables program evaluators to engage instructors in a participatory approach to improving practice, based on item-by-item comparisons, and to improve and maintain the quality of curriculum delivery. The approach is not limited to any specific curriculum and may be broadly applied to curriculum delivery programs in which the instructors and researchers combine to achieve quality improvement.

## Data availability statement

The raw data supporting the conclusions of this article will be made available by the authors, without undue reservation.

## Ethics statement

Ethical review and approval was received through an IRB human subjects research determination finding IRB was not required for the study on human participants in accordance with the local legislation and institutional requirements. The legal guardian/next of kin provided their informed consent for participation in this study.

## Author contributions

Study conception, design, analysis and interpretation of results, and draft manuscript preparation: GG, RL, and BR. Instrument validation and data collection: GG and BR. All authors wrote, reviewed the results and approved the final version of the manuscript.

## Funding

Publication of this article was supported by Cooperative Agreement # TP2AH000059-01-00 for the Project Titled Equipping Youth with Powerful Choices by the Department of Health and Human Services, Office of Population Health and Equipping Youth Inc., Cedar Rapids, Iowa, with additional funding support by the Administration for Children Youth and Families-Family and Youth Services Bureau Grant # 90SR0046-01-00: Risk Avoidance Education for Iowa's Youth.

## Conflict of interest

The authors declare that the research was conducted in the absence of any commercial or financial relationships that could be construed as a potential conflict of interest.

## Publisher's note

All claims expressed in this article are solely those of the authors and do not necessarily represent those of their affiliated organizations, or those of the publisher, the editors and the reviewers. Any product that may be evaluated in this article, or claim that may be made by its manufacturer, is not guaranteed or endorsed by the publisher.

## Footnotes

1 The action planning step in the model is similar to process improvement methods such as before and after action reviews, AARs and BARs: the Plan-Do-Study-Act model, Implementation Science, Six Sigma, or any of several other process improvement methods that involve group decisions for next steps; however, unlike this model, those do not begin with systematically gathered longitudinal data.
