# Supplementary material for: Improving curriculum delivery: Using a results informed quality improvement model for teen behavioral health education
Source: Front Public Health. 2022 Nov 16;10:965534. doi: 10.3389/fpubh.2022.965534 (PMC9709193; doi:10.3389/fpubh.2022.965534)
Supplement: Supplementary file 1 [file Data_Sheet_1.docx]

**Appendix 1**

**Powerful Choices CQI Report for 2020 Quarter 1**

**(January, 2020)**

|  |  | *When I need to make an important decision I would?* | | |  |  |
| --- | --- | --- | --- | --- | --- | --- |
|  | **Follow-up** | | | | |  |
| **Initial** |  | **A.** | **B.** | **C.** | **D.** | **Initial Totals** |
| **A.Ask my friends.** | Count | 10 | 1 | 12 | 4 | 27 |
|  | % of Total | 5.7% | 0.6% | 6.8% | 2.3% | 15.3% |
| **B.Google the answer.** | Count | 0 | 2 | 8 | 2 | 12 |
|  | % of Total | 0.0% | 1.1% | 4.5% | 1.1% | 6.8% |
| **C.Ask a trusted adult.** | Count | 4 | 0 | 88 | 9 | 101 |
|  | % of Total | 2.3% | 0.0% | 50.0% | 5.1% | 57.4% |
| **D.Think about it and wait and see what happens.** | Count | 4 | 0 | 20 | 12 | 36 |
|  | % of Total | 2.3% | 0.0% | 11.4% | 6.8% | 20.5% |
| **Follow-up Totals** | Count | 18 | 3 | 128 | 27 | 176 |
|  | % of Total | 10.2% | 1.7% | 72.7% | 15.3% | 100.0% |

The follow-up assessment obtained 72.7% correct compared to the initial 57.4% correct for choice “C”, the correct response. “D” was selected by 20.5% of students initially and by 15.3% at follow-up; “A” was selected initially by 15.3% and by 10.2% at follow-up. These results indicate 25.5% indicate either a. “ask my friends” or d. “think about it and wait and see what happens” remained relatively frequent incorrect responses.

|  |  | *Gaining positive relationships is experienced best by?* | | | |  |
| --- | --- | --- | --- | --- | --- | --- |
| **Initial** |  | **Follow-up** | | | | **Initial Totals** |
|  |  | **A.** | **B.** | **C.** | **D.** |  |
| **A.Joining a gym for working out.** | Count | 3 | 2 | 6 | 0 | 11 |
|  | % of Total | 1.7% | 1.1% | 3.4% | 0.0% | 6.2% |
| **B.Getting a job** | Count | 0 | 9 | 11 | 0 | 20 |
|  | % of Total | 0.0% | 5.1% | 6.2% | 0.0% | 11.2% |
| **C.Working together to help others.** | Count | 1 | 12 | 129 | 2 | 144 |
|  | % of Total | 0.6% | 6.7% | 72.5% | 1.1% | 80.9% |
| **D.Club membership** | Count | 0 | 2 | 1 | 0 | 3 |
|  | % of Total | 0.0% | 1.1% | 0.6% | 0.0% | 1.7% |
| **Follow-up Totals** | Count | 4 | 25 | 147 | 2 | 178 |
|  | % of Total | 2.2% | 14.0% | 82.6% | 1.1% | 100.0% |

The follow-up assessment obtained 82.6% correct compared to the initial 80.9% correct for choice “C”, the correct response. Notably, 14.0% of students selected choice “B” incorrectly at follow-up compared to 11.2% of students at initial assessment. Twelve (12) students who selected the correct answer “C” on the initial assessment selected “B”, “getting a job” on the follow-up.

|  |  | *The best way to develop a close friendship is?* | | | |  |
| --- | --- | --- | --- | --- | --- | --- |
|  | **Follow-up** | | | | | **Initial Totals** |
| **Initial** |  | **A.** | **B.** | **C.** | **D.** |  |
| **A.Finding someone who will to spend a lot of time with me.** | Count | 20 | 18 | 0 | 2 | 40 |
|  | % of Total | 11.2% | 10.1% | 0.0% | 1.1% | 22.5% |
| **B.Finding someone who helps me make positive choices.** | Count | 10 | 82 | 0 | 5 | 97 |
|  | % of Total | 5.6% | 46.1% | 0.0% | 2.8% | 54.5% |
| **C.Finding someone when playing games I like online.** | Count | 1 | 5 | 4 | 1 | 11 |
|  | % of Total | 0.6% | 2.8% | 2.2% | 0.6% | 6.2% |
| **D.Spend time with others after sports or activities at school.** | Count | 3 | 20 | 1 | 6 | 30 |
|  | % of Total | 1.7% | 11.2% | 0.6% | 3.4% | 16.9% |
| **Follow-up Totals** | Count | 34 | 125 | 5 | 14 | 178 |
|  | % of Total | 19.1% | 70.2% | 2.8% | 7.9% | 100.0% |

The follow-up assessment obtained 70.2% correct compared to the initial 54.5% correct for choice “B”, the correct response. Forty students selected choice “A” at initial assessment and 34 students (19.1%) selected “A” at follow-up; 20 students chose “A” on the initial and follow-up assessment.

|  |  | *How do I avoid behavior that could have a negative consequence for me?* | | | |  |
| --- | --- | --- | --- | --- | --- | --- |
| **Initial** |  | **Follow-up** | | | | **Initial Totals** |
|  |  | **A.** | **B.** | **C.** | **D.** |  |
| **A.Make boundaries for myself.** | Count | 55 | 2 | 2 | 4 | 63 |
|  | % of Total | 30.9% | 1.1% | 1.1% | 2.2% | 35.4% |
| **B.Stand up for what I feel is right.** | Count | 32 | 4 | 2 | 4 | 42 |
|  | % of Total | 18.0% | 2.2% | 1.1% | 2.2% | 23.6% |
| **C.Avoid people who compromise my values.** | Count | 20 | 2 | 1 | 3 | 26 |
|  | % of Total | 11.2% | 1.1% | 0.6% | 1.7% | 14.6% |
| **D.Consider what happens because of what I do.** | Count | 37 | 3 | 1 | 6 | 47 |
|  | % of Total | 20.8% | 1.7% | 0.6% | 3.4% | 26.4% |
| **Follow-up Totals** | Count | 144 | 11 | 6 | 17 | 178 |
|  | % of Total | 80.9% | 6.2% | 3.4% | 9.6% | 100.0% |

The follow-up assessment obtained 80.9% correct compared to the initial 35.4% correct for choice “A”, the correct response. The dramatic improvement in the correct responses at follow-up accompany consistently low frequencies of incorrect responses across all incorrect response options at follow-up demonstrating a highly desirable example of improvement and effective curriculum delivery.

|  | *What makes people successful in life?* | | | | |  |
| --- | --- | --- | --- | --- | --- | --- |
| ***Initial*** |  | ***Follow-up*** | | | | ***Initial Totals*** |
|  |  | **A.** | **B.** | **C.** | **D.** |  |
| **A.Supportive parent/caregiver.** | Count | 4 | 13 | 0 | 0 | 17 |
|  | % of Total | 2.2% | 7.3% | 0.0% | 0.0% | 9.6% |
| **B.Setting goals.** | Count | 6 | 124 | 0 | 4 | 134 |
|  | % of Total | 3.4% | 69.7% | 0.0% | 2.2% | 75.3% |
| **C.Having money.** | Count | 1 | 6 | 4 | 0 | 11 |
|  | % of Total | 0.6% | 3.4% | 2.2% | 0.0% | 6.2% |
| **D.Being intelligent.** | Count | 1 | 11 | 1 | 3 | 16 |
|  | % of Total | 0.6% | 6.2% | 0.6% | 1.7% | 9.0% |
| **Follow-up Totals** | Count | 12 | 154 | 5 | 7 | 178 |
|  | % of Total | 6.7% | 86.5% | 2.8% | 3.9% | 100.0% |

The follow-up assessment obtained 86.5% correct compared to the initial 75.3% correct for choice “B”, the correct response.

|  |  | *How can you avoid bad habits?* | | |  |  |
| --- | --- | --- | --- | --- | --- | --- |
| **Initial** | **Follow-up** | | | |  | **Initial Totals** |
|  |  | **A.** | **B.** | **C.** | **D.** |  |
| **A.Never start doing or using something that is habit forming.** | Count | 138 | 2 | 1 | 0 | 141 |
|  | % of Total | 77.5% | 1.1% | 0.6% | 0.0% | 79.2% |
| **B.Stop when you know you are in trouble.** | Count | 26 | 1 | 1 | 0 | 28 |
|  | % of Total | 14.6% | 0.6% | 0.6% | 0.0% | 15.7% |
| **C.Try it and then quit.** | Count | 2 | 0 | 3 | 0 | 5 |
|  | % of Total | 1.1% | 0.0% | 1.7% | 0.0% | 2.8% |
| **D.Quit the habit when parents find out.** | Count | 3 | 1 | 0 | 0 | 4 |
|  | % of Total | 1.7% | 0.6% | 0.0% | 0.0% | 2.2% |
| **Follow-up Totals** | Count | 169 | 4 | 5 | 0 | 178 |
|  | % of Total | 94.9% | 2.2% | 2.8% | 0.0% | 100.0% |

The follow-up assessment obtained 94.9% correct compared to the initial 79.2% correct for choice “A”, the correct response. Among the 15.7% who initially chose “B”, only 2.2% chose “B” at follow-up suggesting highly effective curriculum delivery for this content area.

| *What would you do if a person from your class spread a rumor about your close friend on social media?* | | | | | | |
| --- | --- | --- | --- | --- | --- | --- |
| **Initial** | **Follow-up** | | | |  | **Initial Totals** |
|  |  | **A.** | **B.** | **C.** | **D.** |  |
| **A.Confront them and threaten them if they don't stop.** | Count | 21 | 3 | 2 | 14 | 40 |
|  | % of Total | 11.8% | 1.7% | 1.1% | 7.9% | 22.5% |
| **B.Ignore the negative rumor.** | Count | 3 | 18 | 0 | 19 | 40 |
|  | % of Total | 1.7% | 10.1% | 0.0% | 10.7% | 22.5% |
| **C.Post negative rumors about the person who started it.** | Count | 1 | 0 | 2 | 3 | 6 |
|  | % of Total | 0.6% | 0.0% | 1.1% | 1.7% | 3.4% |
| **D.Tell other classmates to help me post and tell what is true about my friend.** | Count | 3 | 10 | 0 | 79 | 92 |
|  | % of Total | 1.7% | 5.6% | 0.0% | 44.4% | 51.7% |
| **Follow-up Totals** | Count | 28 | 31 | 4 | 115 | 178 |
|  | % of Total | 15.7% | 17.4% | 2.2% | 64.6% | 100.0% |

The follow-up assessment obtained 64.6% correct compared to the initial 51.7% correct for choice “D”, the correct response. “A” was selected by 15.7% at follow-up compared to 22.5% initially, and “B” was selected by 17.4% follow-up compared to 22.5% initially. Combined, “A” and “B” represent 33.1 % of all responses indicating this content area is one that could benefit from examination of ways to improve curriculum delivery.

|  |  | *Wisdom is best gained by which of the following?* | | | |  |
| --- | --- | --- | --- | --- | --- | --- |
| **Initial** | **Follow-up** | | | | | **Initial Totals** |
|  |  | **A.** | **B.** | **C.** | **D.** |  |
| **A.Having a good IQ score.** | Count | 4 | 1 | 0 | 3 | 8 |
|  | % of Total | 2.2% | 0.6% | 0.0% | 1.7% | 4.5% |
| **B.Watching YouTube.** | Count | 0 | 3 | 0 | 2 | 5 |
|  | % of Total | 0.0% | 1.7% | 0.0% | 1.1% | 2.8% |
| **C.Getting a great deal of education.** | Count | 2 | 0 | 2 | 22 | 26 |
|  | % of Total | 1.1% | 0.0% | 1.1% | 12.4% | 14.6% |
| **D.Having insight from knowledge, understanding and experience.** | Count | 1 | 0 | 2 | 136 | 139 |
|  | % of Total | 0.6% | 0.0% | 1.1% | 76.4% | 78.1% |
| **Follow-up Totals** | Count | 7 | 4 | 4 | 163 | 178 |
|  | % of Total | 3.9% | 2.2% | 2.2% | 91.6% | 100.0% |

The follow-up assessment obtained 91.6% correct compared to the initial 78.1% correct for choice “D”, the correct response.

|  | *According to Powerful Choices lessons, when is the best time for a person to become sexually active?* | | | | | |
| --- | --- | --- | --- | --- | --- | --- |
| **Initial** | **Follow-up** | | | | | **Initial Totals** |
|  |  | **A.** | **B.** | **C.** | **D.** |  |
| **A. When they are married.** | Count | 35 | 3 | 8 | 1 | 47 |
|  | % of Total | 19.8% | 1.7% | 4.5% | 0.6% | 26.6% |
| **B. When they think they are in love.** | Count | 8 | 6 | 7 | 2 | 23 |
|  | % of Total | 4.5% | 3.4% | 4.0% | 1.1% | 13.0% |
| **C. To satisfy their curiosity.** | Count | 10 | 3 | 10 | 1 | 24 |
|  | % of Total | 5.6% | 1.7% | 5.6% | 0.6% | 13.6% |
| **D. When they think they are ready.** | Count | 29 | 6 | 29 | 19 | 83 |
|  | % of Total | 16.4% | 3.4% | 16.4% | 10.7% | 46.9% |
| **Follow-up Totals** | Count | 82 | 18 | 54 | 23 | 177 |
|  | % of Total | 46.3% | 10.2% | 30.5% | 13.0% | 100.0% |

The follow-up assessment obtained 46.3% correct compared to the initial 26.6% correct for choice “A”, the correct response. However, this represents a low threshold of correct responses since less than half of students chose the correct answer at follow-up. While 13.6% selected “C” initially, 30.5% selected “C” at follow-up. Examining this increase in the percent incorrectly responding “C” is important for improving curriculum delivery in this content area.

|  | *Accepting the challenge of thinking before acting helps a person to?* | | | | |  |
| --- | --- | --- | --- | --- | --- | --- |
| **Initial** | **Follow-up** | | | | | **Initial Totals** |
|  |  | **A.** | **B.** | **C.** | **D.** |  |
| **A.Have time to ask my friends for advice.** | Count | 2 | 10 | 0 | 4 | 16 |
|  | % of Total | 1.1% | 5.7% | 0.0% | 2.3% | 9.1% |
| **B. Get the best revenge.** | Count | 0 | 18 | 0 | 7 | 25 |
|  | % of Total | 0.0% | 10.3% | 0.0% | 4.0% | 14.3% |
| **C. Never make a mistake.** | Count | 5 | 2 | 2 | 5 | 14 |
|  | % of Total | 2.9% | 1.1% | 1.1% | 2.9% | 8.0% |
| **D.Avoid the result of doing something risky.** | Count | 4 | 37 | 4 | 75 | 120 |
|  | % of Total | 2.3% | 21.1% | 2.3% | 42.9% | 68.6% |
| **Follow-up Totals** | Count | 11 | 67 | 6 | 91 | 175 |
|  | % of Total | 6.3% | 38.3% | 3.4% | 52.0% | 100.0% |

The follow-up assessment obtained 52% correct compared to the initial 68.6% correct for choice “D”, the correct response. At initial assessment 14.3% selected the incorrect choice “B”, and at follow-up the percentage more than doubled to 38.3% choosing “B.” This content area requires attention to discover what is needed to more effectively deliver this content area.

|  | *How can you build a strong foundation for your life?* | | | | |  |
| --- | --- | --- | --- | --- | --- | --- |
| **Initial** | **Follow-up** | | | | | **Initial Totals** |
|  |  | **A.** | **B.** | **C.** | **D.** |  |
| **A.Always do what feels the best for you.** | Count | 35 | 1 | 58 | 5 | 99 |
|  | % of Total | 19.9% | 0.6% | 33.0% | 2.8% | 56.3% |
| **B.Do what other people are doing.** | Count | 2 | 1 | 0 | 0 | 3 |
|  | % of Total | 1.1% | 0.6% | 0.0% | 0.0% | 1.7% |
| **C.Make and commit to keeping my boundaries.** | Count | 6 | 0 | 47 | 2 | 55 |
|  | % of Total | 3.4% | 0.0% | 26.7% | 1.1% | 31.3% |
| **D.Wait until I am older to make important decisions.** | Count | 4 | 0 | 14 | 1 | 19 |
|  | % of Total | 2.3% | 0.0% | 8.0% | 0.6% | 10.8% |
| **Follow-up Totals** | Count | 47 | 2 | 119 | 8 | 176 |
|  | % of Total | 26.7% | 1.1% | 67.6% | 4.5% | 100.0% |

The follow-up assessment obtained 67.6% correct compared to the initial 31.3% correct for choice “C”, the correct response. While the percentage selecting “A” was initially 56.3% and decreased to 26.7% at follow-up, the 26.7% indicates a need for improvement in curriculum delivery for this content area.

|  | *The use of drugs and alcohol have the strongest effect on?* | | | | |  |
| --- | --- | --- | --- | --- | --- | --- |
| **Initial** | **Follow-up** | | | | | **Initial Totals** |
|  |  | **A.** | **B.** | **C.** | **D.** |  |
| **A.Excelling at sports or activities.** | Count | 0 | 6 | 0 | 1 | 7 |
|  | % of Total | 0.0% | 3.4% | 0.0% | 0.6% | 4.0% |
| **B.Decision making.** | Count | 2 | 59 | 1 | 18 | 80 |
|  | % of Total | 1.1% | 33.5% | 0.6% | 10.2% | 45.5% |
| **C.Success in school.** | Count | 1 | 1 | 1 | 3 | 6 |
|  | % of Total | 0.6% | 0.6% | 0.6% | 1.7% | 3.4% |
| **D.Having a healthy lifestyle.** | Count | 0 | 45 | 3 | 35 | 83 |
|  | % of Total | 0.0% | 25.6% | 1.7% | 19.9% | 47.2% |
| **Follow-up Totals** | Count | 3 | 111 | 5 | 57 | 176 |
|  | % of Total | 1.7% | 63.1% | 2.8% | 32.4% | 100.0% |

The follow-up assessment obtained 63.1% correct compared to the initial 45.5% correct for choice “B”, the correct response. The percentage of students selecting the incorrect choice “D” was initially 47.2% but at follow-up 32.4% chose “D”. Among those choosing “D” at follow-up are 18 (10.2%) who chose the correct response at initial assessment.

|  | *What does a person need to do to make good decisions for the future?* | | | | |  |
| --- | --- | --- | --- | --- | --- | --- |
| **Initial** | **Follow-up** | | | | | **Initial Totals** |
|  |  | **A.** | **B.** | **C.** | **D.** |  |
| **A.Ask an adult for their opinion.** | Count | 6 | 2 | 0 | 8 | 16 |
|  | % of Total | 3.4% | 1.1% | 0.0% | 4.6% | 9.1% |
| **B.Consider what has happened to friends or family.** | Count | 4 | 3 | 3 | 4 | 14 |
|  | % of Total | 2.3% | 1.7% | 1.7% | 2.3% | 8.0% |
| **C.Experience as much as you can before making a decision.** | Count | 2 | 2 | 11 | 13 | 28 |
|  | % of Total | 1.1% | 1.1% | 6.3% | 7.4% | 16.0% |
| **D.Consider the positive & negative consequences of my choices.** | Count | 6 | 5 | 16 | 90 | 117 |
|  | % of Total | 3.4% | 2.9% | 9.1% | 51.4% | 66.9% |
| **Follow-up Totals** | Count | 18 | 12 | 30 | 115 | 175 |
|  | % of Total | 10.3% | 6.9% | 17.1% | 65.7% | 100.0% |

The follow-up assessment obtained 65.7% correct compared to the initial 66.9% correct for choice “B”, the correct response. Two incorrect choices were selected more frequently follow-up than at initial assessment: choice “A” was selected by 10.3% compared to the initial 9.1%, and choice “C” was selected by 17.1% compared to the initial 16%.. Addressing these two incorrect responses is an opportunity to improve results from the curriculum delivery for this content area.

|  | *Making a good decision is a result of?* | | | | |  |
| --- | --- | --- | --- | --- | --- | --- |
| **Initial** | **Follow-up** | | | | | **Initial Totals** |
|  |  | **A.** | **B.** | **C.** | **D.** |  |
| **A.Doing what you have decided is the very best for you.** | Count | 111 | 13 | 4 | 6 | 134 |
|  | % of Total | 63.1% | 7.4% | 2.3% | 3.4% | 76.1% |
| **B.Talking to friends that have experienced the same things.** | Count | 9 | 5 | 1 | 2 | 17 |
|  | % of Total | 5.1% | 2.8% | 0.6% | 1.1% | 9.7% |
| **C.Going to school and listening to your teacher.** | Count | 10 | 1 | 1 | 1 | 13 |
|  | % of Total | 5.7% | 0.6% | 0.6% | 0.6% | 7.4% |
| **D.Doing what your parents tell you to do.** | Count | 6 | 3 | 0 | 3 | 12 |
|  | % of Total | 3.4% | 1.7% | 0.0% | 1.7% | 6.8% |
| **Follow-up Totals** | Count | 136 | 22 | 6 | 12 | 176 |
|  | % of Total | 77.3% | 12.5% | 3.4% | 6.8% | 100.0% |

The follow-up assessment obtained 77.3% correct compared to the initial 76.1% correct for choice “A”, the correct response. The number of students selecting the incorrect choice “B” was initially 9.7% and increased to 12.5% at follow-up.

|  | *Taking Powerful Choices lessons results in?* | | | | |  |
| --- | --- | --- | --- | --- | --- | --- |
| **Initial** | **Follow-up** | | | | | **Initial Totals** |
|  |  | **A.** | **B.** | **C.** | **D.** |  |
| **A.More knowledge for advising my friends** | Count | 11 | 9 | 0 | 1 | 21 |
|  | % of Total | 6.2% | 5.1% | 0.0% | 0.6% | 11.8% |
| **B.Stopping to think before making a choice** | Count | 11 | 123 | 1 | 3 | 138 |
|  | % of Total | 6.2% | 69.1% | 0.6% | 1.7% | 77.5% |
| **C.Not getting caught when I do wrong** | Count | 0 | 7 | 4 | 0 | 11 |
|  | % of Total | 0.0% | 3.9% | 2.2% | 0.0% | 6.2% |
| **D.Knowing how to get even when someone offends me** | Count | 2 | 5 | 0 | 1 | 8 |
|  | % of Total | 1.1% | 2.8% | 0.0% | 0.6% | 4.5% |
| **Follow-up Totals** | Count | 24 | 144 | 5 | 5 | 178 |
|  | % of Total | 13.5% | 80.9% | 2.8% | 2.8% | 100.0% |

The follow-up assessment obtained 80.9% correct compared to the initial 77.5% correct for choice “B”, the correct response. The number of students selecting the incorrect choice “A” was initially 11.8% and at follow-up the percentage choosing “A” was 13.5%; other incorrect responses were less than 3%.

|  | *To have the most successful life a person should?* | | | | |  |
| --- | --- | --- | --- | --- | --- | --- |
| **Initial** | **Follow-up** | | | | | **Initial Totals** |
|  |  | **A.** | **B.** | **C.** | **D.** |  |
| **A.Finish high school, get a good job, not have children before marriage.** | Count | 80 | 4 | 1 | 0 | 85 |
|  | % of Total | 44.9% | 2.2% | 0.6% | 0.0% | 47.8% |
| **B.Remain single, go to college, get a good job.** | Count | 11 | 6 | 2 | 1 | 20 |
|  | % of Total | 6.2% | 3.4% | 1.1% | 0.6% | 11.2% |
| **C.Get a good job, pay all bills on time.** | Count | 21 | 0 | 3 | 3 | 27 |
|  | % of Total | 11.8% | 0.0% | 1.7% | 1.7% | 15.2% |
| **D.Get a job that I like and that pays well.** | Count | 34 | 1 | 0 | 11 | 46 |
|  | % of Total | 19.1% | 0.6% | 0.0% | 6.2% | 25.8% |
| **Follow-up Totals** | Count | 146 | 11 | 6 | 15 | 178 |
|  | % of Total | 82.0% | 6.2% | 3.4% | 8.4% | 100.0% |

The follow-up assessment obtained 82% correct compared to the initial 47.8% correct for choice “A”, the correct response. The dramatic improvement in the correct responses at follow-up accompany consistently low frequencies of incorrect responses across all incorrect response options at follow-up demonstrating an example of desired improvement and suggests effective curriculum delivery.

|  | *How do you show your friend that you care when they make mistakes?* | | | | |  |
| --- | --- | --- | --- | --- | --- | --- |
| **Initial** | **Follow-up** | | | | | **Initial Totals** |
|  |  | **A.** | **B.** | **C.** | **D.** |  |
| **A.Tell them what they should have done.** | Count | 50 | 2 | 1 | 21 | 74 |
|  | % of Total | 28.4% | 1.1% | 0.6% | 11.9% | 42.0% |
| **B.Don't talk about their mistakes with them.** | Count | 5 | 0 | 0 | 2 | 7 |
|  | % of Total | 2.8% | 0.0% | 0.0% | 1.1% | 4.0% |
| **C.Advise them not to think about it.** | Count | 5 | 1 | 4 | 8 | 18 |
|  | % of Total | 2.8% | 0.6% | 2.3% | 4.5% | 10.2% |
| **D.Listen without passing judgment or telling others.** | Count | 19 | 3 | 1 | 54 | 77 |
|  | % of Total | 10.8% | 1.7% | 0.6% | 30.7% | 43.8% |
| **Follow-up Totals** | Count | 79 | 6 | 6 | 85 | 176 |
|  | % of Total | 44.9% | 3.4% | 3.4% | 48.3% | 100.0% |

The follow-up assessment obtained 48.3% correct compared to the initial 43.8% correct for choice “D”, the correct response. Additionally, this represents a low threshold of correct responses since less than half of students chose the correct answer at follow-up. The number of students selecting the incorrect choice “A” was initially 42% and increased to 44.9% at follow-up. How this content area is delivered is in need of examination to understand how so many chose “A” which is not the correct response. .

|  | *How can you be a positive role model?* | | | | |  |
| --- | --- | --- | --- | --- | --- | --- |
| **Initial** | **Follow-up** | | | | | **Initial Totals** |
|  |  | **A.** | **B.** | **C.** | **D.** |  |
| **A.By making others do what I think is right.** | Count | 43 | 1 | 11 | 8 | 63 |
|  | % of Total | 24.2% | 0.6% | 6.2% | 4.5% | 35.4% |
| **B.By learning from other students.** | Count | 2 | 2 | 6 | 3 | 13 |
|  | % of Total | 1.1% | 1.1% | 3.4% | 1.7% | 7.3% |
| **C.By knowing and acting on what I value.** | Count | 13 | 5 | 44 | 9 | 71 |
|  | % of Total | 7.3% | 2.8% | 24.7% | 5.1% | 39.9% |
| **D.By excelling in all I do.** | Count | 5 | 0 | 14 | 12 | 31 |
|  | % of Total | 2.8% | 0.0% | 7.9% | 6.7% | 17.4% |
| **Follow-up Totals** | Count | 63 | 8 | 75 | 32 | 178 |
|  | % of Total | 35.4% | 4.5% | 42.1% | 18.0% | 100.0% |

The follow-up assessment obtained 42.1% correct compared to the initial 39.9% correct for choice “C”, the correct response. This item also represents a low threshold of correct responses since less than half of students chose the correct answer at follow-up. Importantly, two incorrect choices were selected as frequently at follow-up as at initial assessment. Choice “A” was selected by 35.4% at initial and follow-up, and choice “D” was selected by 18% and 17.4% at follow-up and initial assessment, respectively. Addressing these two incorrect responses is an opportunity to improve curriculum delivery for this content area.
